# Novel Chitin Deacetylase from *Thalassiosira weissflogii* Highlights the Potential for Chitin Derivative Production

**DOI:** 10.3390/metabo13030429

**Published:** 2023-03-15

**Authors:** Mengzhen Cheng, Zhanru Shao, Xin Wang, Chang Lu, Shuang Li, Delin Duan

**Affiliations:** 1 CAS and Shandong Province Key Laboratory of Experimental Marine Biology, Center for Ocean Mega-Science, Institute of Oceanology, Chinese Academy of Sciences, Qingdao 266071, China; 2 Laboratory for Marine Biology and Biotechnology, Qingdao National Laboratory for Marine Science and Technology, Qingdao 266237, China; 3 University of Chinese Academy of Sciences, Beijing 100049, China; 4 Department of Biological Engineering, College of Life Science, Yantai University, Yantai 264005, China

**Keywords:** β-chitin, chitin deacetylase, chitin derivatives, chitinolytic activity, diatom, enzyme and product structure

## Abstract

β-Chitin is an important carbon fixation product of diatoms, and is the most abundant nitrogen-containing polysaccharide in the ocean. It has potential for widespread application, but the characterization of chitin-related enzymes from β-chitin producers has rarely been reported. In this study, a chitin deacetylase (TwCDA) was retrieved from the Marine Microbial Eukaryote Transcriptome Sequencing Project (MMETSP) database and was heterologously expressed in vitro for functional analysis. The results showed that both the full-length sequence (TwCDA) and the *N*-terminal truncated sequence (TwCDA-S) had chitin deacetylase and chitinolytic activities after expression in *Escherichia coli*. High-performance liquid chromatography (HPLC) and gas chromatography–mass spectrometry (GC-MS) indicated that TwCDA and TwCDA-S could catalyze the deacetylation of oligosaccharide (GlcNAc)_5_. TwCDA had higher deacetylase activity, and also catalyzed the deacetylation of the β-chitin polymer. A dinitrosalicylic acid (DNS) assay showed that TwCDA-S had high chitinolytic activity for (GlcNAc)_5_, and the optimal reaction temperature was 35 °C. Liquid chromatography combined with time-of-flight mass spectrometry (LC-coTOF-MS) detected the formation of a *N*-acetylglucosamine monomer (C_8_H_15_NO_6_) in the reaction mixture. Altogether, we isolated a chitin deacetylase from a marine diatom, which can catalyze the deacetylation and degradation of chitin and chitin oligosaccharides. The relevant results lay a foundation for the internal regulation mechanism of chitin metabolism in diatoms and provide a candidate enzyme for the green industrial preparation of chitosan and chitin oligosaccharides.

## 1. Introduction

Chitin, β-(1,4)-linked *N*-acetylglucosamine (GlcNAc) is the most abundant renewable polymer in the ocean [1,2]. It is an important source of carbon and nitrogen in marine environments, where billions of tons are produced each year [3]. Its natural reserves are second only to those of cellulose, widely found in marine mollusks, crustaceans, and diatoms [4]. Chitin has three crystal polymorphs, α, β, and γ [5]. The chain segments of β-chitin are arranged in parallel, which is more accessible to produce amorphous structures. β-Chitin exhibits better reactivity and solubility, and has potential to produce derivatives with higher bioactivities [6,7]. Previous reports showed that β-chitin only exists in marine molluscs (such as squid) and some diatoms [8,9]. Diatoms are the most abundant group of eukaryotic microalgae, which contribute up to 40% of marine primary productivity [10,11,12]. Chitin fibers extend from the theca through the fultoportulae, and have been reported to represent approximately 30% of the fresh weight of diatom cells [13]. *Thalassiosira* and *Cyclotella* in centric diatoms are the most important producers of marine chitin [14,15]. Through a cell wall polysaccharide glycosidic linkage analysis, Cheng et al. [16] found that, of the three typical species of *Thalassiosira* (*T. pseudonana*, *T. rotula*, and *T. weissflogii*), the chitin content in the cell wall of *T. weissflogii* was the highest, indicating that *T. weissflogii* might have a strong ability to synthesize chitin and is an excellent material for the exploration of chitin-related enzyme resources and chitin derivatives.

Chitin and its derivatives are used in medicine, pharmaceuticals, cosmetics, the food industry, agriculture, environmental protection, etc. However, due to the strong hydrophobicity of chitin, its application is greatly limited, and its economic value has not been fully appreciated [17]. In contrast, many chitin derivatives, such as chitosan and chitooligosaccharide, have better physicochemical and physiological properties, especially solubility and biocompatibility [18]. Chitosan, also known as soluble chitin, is derived from chitin by the catalyzation of chitin deacetylase [19,20]. Chitooligosaccharides are oligomers of chitosan that are produced by the decomposition of chitosan by chitinase [20,21].

Chitin deacetylase (CDA, E.C.3.5.1.41) belongs to the carbohydrate esterase 4 (CE4) superfamily. It participates in the degradation pathway of chitin and has a wide range of substrate specificity. It can catalyze the deacetylation of both chitin oligosaccharides and insoluble chitin [22]. The initial study of chitin deacetylase focused on screening bacteria and fungal strains producing CDA [23,24]. Subsequently, CDA genes were extensively identified and isolated, facilitating the exploration of their enzymatic activities [25,26]. In insects, CDA has been confirmed to play an important role in morphogenesis and the immune response [27,28,29]. In bacteria and fungi, CDAs may contribute to chitin degradation for nutritional purposes, and most research has focused on cloning the CDA genes and identifying their enzyme activities [30,31,32]. It has also been reported that a few pathogenic fungal CDA can render the fungus less susceptible to host immunosurveillance [33]. Recently, RNA interference (RNAi) and gene knockout have also been applied to study the function of chitin deacetylase. Yang et al. [34] characterized four CDAs from *Sogatella furcifera* and found that three of them were associated with molting defects and high mortality with nymph–adult molting. Li et al. [35] knocked out the *EScDA-l* gene in *Eriocheir sinensis*, which resulted in an abnormal ultrastructure of the cuticle, suggesting that *EsCDA-l* is indispensable for molting. However, present research is still largely restricted to bacteria, fungi, and crustaceans, and there have been few reports of chitin deacetylase in eukaryotic microalgae. To our knowledge, the only report on functional CDA in diatoms noted the overexpression of two diatom CDA genes (*PtCDA* and *TpCDA*) in *Phaeodactylum tricornutum* to address the ambiguous characteristics of CDAs from *P. tricornutum* and *T. pseudonana* [36].

In this study, a putative *CDA* (*TwCDA*) was identified from *T. weissflogii*, and its sequence structure, evolutionary relationships, and enzymatic activities were integrally characterized. The results verified the role of TwCDA in the degradation of chitin in vitro, which lays a foundation for determining the underlying regulation mechanism of chitin metabolism in diatoms, and they provide a new candidate enzyme for the preparation of chitosan and chitooligosaccharides.

## 2. Materials and Methods

### 2.1. Sample Culture

*T. weissflogii* (9021) was acquired from the Microalgae Collection Center at Ningbo University, Ningbo, China, and grown in an optimized f/2 liquid medium provided by Shanghai Guangyu Biological Technology Co., Ltd. (Shanghai, China) (Table S1). Cells were cultured at 20 °C in 12 h:12 h light:dark diurnal cycles (100 µmol m^−2^ s^−1^) with shaking at 100× rpm. Cells at the exponential phase were collected by centrifugation, flash-frozen in liquid nitrogen, and stored at −80 °C for further experiments.

### 2.2. Substrate Preparation

Thus far, there is no pure beta-chitin standard for sale on the market. β-Chitin was extracted from *T. weissflogii* by a method optimized from the report by Noishiki et al. [37]. The cells were collected by centrifugation at 2000× *g*. The supernatant and pellets were decolorized by adding methanol (65 °C, 2 h), deproteinized with 5% KOH (room temperature, overnight), demineralized with 0.34% NaClO_2_ (70 °C, 6 h) and 0.1 N HCl (boiling, 1 h), and purified with 1% HF (room temperature, overnight). At the end of each step, the sample was centrifuged at 19,000× *g* to remove the supernatant, and finally it was dehydrated at 80 °C and stored at −80 °C [37]. An α-chitin standard product was purchased from Macklin (Shanghai, China). The crystalline structure of the extracted chitin and α-chitin standard were detected by infrared spectra (Thermo Fisher Scientific, IS50, Waltham, MA, USA). Chitin oligomers (pentamer) were kindly supplied by the laboratory of Prof. Bruno M. Moerschbacher, University of Münster, Germany.

### 2.3. Cloning and Sequence Analysis of TwCDA

Total RNA was extracted by grinding the *T. weissflflogii* frozen sample in TRIzol reagent (Life Technologies, Carlsbad, CA, USA). Then, 1.5 mL TRIzol reagent was added to the frozen algal pellet; it was placed at room temperature for 5 min and centrifuged at 10,000× rpm, at 4 °C. The supernatant was added to a new 2 mL tube with 300 μL chloroform and incubated at room temperature for 10 min prior to centrifuging at 10,000× rpm for 5 min. The supernatant was added with 750 μL isopropyl alcohol and centrifuged at 10,000× rpm, 4 °C, for 10 min. Finally, 500 μL 75% ethanol was added and the supernatant was removed by centrifugation at 8500× rpm at 4 °C to obtain RNA. The extracted RNA was reverse-transcribed into cDNA using a reverse transcription kit (SparkJade, Jinan, China), with 200 μL of a reaction mixture of RNA, gDNA eraser, and 2 × SPARK scriptII RT Plus Master Mix. The mixture was incubated in a thermal cycler with the following protocol: 42 °C for 5 min, 50 °C for 30 min, and 80 °C for 5 min. The synthesized cDNA can be directly used for subsequent PCR amplification.

A full-length *CDA* homologous sequence (CAMPEP_0203574602, *TwCDA*) was retrieved from the *T. weissflogii* transcriptome dataset, using a CDA from *T. pseudonana* (TpCDA) [36] to query against the MMETSP database by BLASTP [38]. The full-length *TwCDA* sequence was synthesized with codons optimized for *E. coli* (Sangon Biotech, Shanghai, China). In order to improve the subsequent heterologous expression, we also removed the transmembrane helices to amplify the *N*-terminal truncated *TwCDA-S*. TwCDA-S-F (5′-CGGGATCCATGGAAGATACCATGGCAGC-3′) and TwCDA-S-R (5′-CCAAGCTTCTCGAGCAGGATCATAGTCG-3′), containing a *Bam*HI and a *Hin*dIII restriction site, respectively, were designed for PCR amplification. A total of 20 μL PCR reaction mixture was used, with 1 μL of each primer, 10 μL of 2 × Phanta Master Mix (Vazyme, Nanjing, China), 2 μL of cDNA template, and 6 μL of ddH_2_O. The PCR amplification procedure was as follows: 95 °C for 30 s; 35 cycles of 95 °C for 10 s, 55 °C for 5 s, and 72 °C for 10 s; and 72 °C for 1 min. The products were sequenced and analyzed using BLASTN.

### 2.4. Bioinformatic Analysis of TwCDA

The TwCDA amino acid sequence was aligned with CDA proteins from *T. pseudonana* and *Cyclotella cryptica* by CLUSTALW (https://www.genome.jp/tools-bin/clustalw, accessed on 17 September 2022). ProtParam was used to predict the physical and chemical parameters (molecular weight and isoelectric point) of TwCDA [39]. The Motif search tool (http://www.genome.jp/tools/motif/, accessed on 27 February 2022) was applied to analyze the motifs in the Pfam and NCBI-CDD databases. EUK-MPLOC and PSORT were jointly used to predict the TwCDA subcellular localization [40,41], with the SignalP v4.1 server (http://www.cbs.dtu.dk/services/SignalP-4.1/, accessed on 27 February 2022) for signal peptide and signal anchoring sequence prediction [42]. TMHMM Server v2.0 (http://www.cbs.dtu.dk/services/TMHMM/, accessed on 27 February 2022) was used to predict transmembrane helices. The secondary structures were illustrated by ESPript [43], and the tertiary structure was predicted by SWISS-MODEL [44]. For phylogenetic analysis, sequences highly homologous to the full-length amino acid sequence of TwCDA were retrieved from three genome resources: MMETSP (https://www.imicrobe.us/#/projects/104, accessed on 10 July 2022), PhycoCosm (https://phycocosm.jgi.doe.gov/phycocosm/home/, accessed on 10 July 2022), and PLAZA (https://bioinformatics.psb.ugent.be/plaza/versions/plaza_diatoms_01/, accessed on 10 July 2022). Homology alignment was conducted using BLASTP. MEGA X was used to construct the neighbor-joining tree based on the Jones–Taylor–Thornton (JTT) model and the Gamma-distributed matrix-based model with 1000 bootstrap replicates [45].

### 2.5. Expression of Recombinant TwCDA Proteins

The pET system was used to express TwCDA and TwCDA-S in vitro. The codon-optimized full-length sequence of *TwCDA* was synthesized into pET-32a vectors with a His-tag (pET-TwCDA, Sangon Biotech, Shanghai, China). At the same time, *TwCDA-S* with a reduced transmembrane helix structure, which was amplified as an *Bam*HI/*Hind*III fragment by PCR, was cloned into the pET-32a vectors with His-tag, resulting in the fusion plasmid pET-TwCDA-S.

The *E. coli* cells (BL21 (DE3) pLysS and Transetta (DE3)) were transformed with the recombinant expression plasmids (pET-TwCDA and pET-TwCDA-S). The *E. coli* cells were then incubated in 1 L of Luria–Bertani (LB) medium with 100 µg/mL of ampicillin and 20 µg/mL of chloramphenicol at 37 °C. When OD_600_ reached 0.6, 0.1 mM IPTG was supplemented, and the induction conditions were changed to 120× rpm and 15 °C. After 24 h of incubation, the transformed cells were harvested by centrifugation at 4500× rpm at 4 °C for 30 min. Then, the cells were disrupted by sonication for 25 min (3 s on and 5 s off cycle) (Xinzhi, Ningbo, China). One EDTA-free protease inhibitor tablet (Roche, Basel, Switzerland) was added to the 35 mL extracts prior to the centrifugation at 12,000× rpm at 4 °C for 45 min. The supernatant was then filtered through a 0.45 μm filter membrane to obtain a crude enzyme solution. The crude protein concentration was measured using a BCA protein quantification kit (Vazyme, Nanjing, China).

### 2.6. SDS-PAGE and Western Blotting

The protein concentration in crude protein was determined with a BCA kit (Solarbio, Beijing, China) according to the manufacturer’s instructions. We then boiled the samples for 10 min in loading dye (125 mM Tris/HCl, 10% SDS, 0.25% BPB, 10% 2-mercaptoethanol, 50% glycerol), and loaded 60 μg of the crude extract onto 12% acrylamide gels for separation by SDS-PAGE. Primary (anti his-tag mouse monoclonal antibody at a concentration of 1:500) and secondary (goat anti-mouse IgG, HRP-conjugated at a concentration of 1:2000) antibodies were used for the Western blotting test via the iBind™Flex Western System (Thermo Fisher Scientific, Waltham, MA, USA) at room temperature for 4 h, following the manufacturer’s protocol. After washing with ddH_2_O solution three times, CDA target bands were visualized by incubating the membranes in ECL reagents with the ChemiDoc XRS Imaging System and the Image Lab 6.0 software (Bio-Rad Lab, Hercules, CA, USA).

### 2.7. Detection of Chitin Deacetylase Activity

Crude protein extracts of TwCDA and TwCDA-S were prepared with a phosphate buffer containing 20 mM sodium phosphate pH 7.5, 20 mM imidazole, 500 mM sodium chloride, 5% glycerol, and a protease inhibitor. The reaction volume was set to 10 mL, containing 50 mM triethanolamine (TEA) pH 7.0, 2 mg/mL substrate, and 1 mL (*c*. 3 mg) crude proteins. The reaction was carried out at 250× *g*, 37 °C for 48 h, and terminated by freezing the mixtures in liquid nitrogen [36]. The inactivated crude enzyme was used as the control group. The substrate specificity was tested towards chitin pentamers (GlcNAc)_5_ and β-chitin. Each experiment was performed with two replicates. Deacetylation activity was determined by measuring the released acetic acid via high-performance liquid chromatography (HPLC) with an acetic acid content detection kit (Solarbio, Beijing, China). The experimental conditions were as follows: an Ultimate AQ-C18 (Yuexu Technology, Shanghai, China) HPLC column (5 µm, 250 × 4.6 mm) was used for detection at 30 °C with a 210 nm UV filter, and the flow rate was set to 0.4 mL/min. Each acetic acid assay was performed with three replicates. A standard curve generated from 175, 87.5, 17.5, 8.75, 1.75, and 0.875 μmol/mL acetic acid was used to calculate the acetic acid concentration in the sample, using Equation (1). Gas chromatography-mass spectrometry (GC-MS) was used to further detect whether acetic acid was produced in the product.

y = 59506x + 72815 (*R*^2^ = 0.9994) (1)

### 2.8. Detection of Chitinolytic Activity

Some chitin deacetylases also have the ability to degrade chitin sugar chains [46]. The dinitrosalicylic acid (DNS) method was used to detect the presence of reducing sugar in the reaction products to verify whether TwCDA has chitinolytic activity. β-Chitin and (GlcNAc)_5_ were used as substrates. The inactivated crude enzyme was used as the control group, and the denatured protein precipitate was removed by centrifugation. The conditions of the enzymatic reaction were as follows: 50 μL of crude enzyme was added to 50 μL of substrate and it was incubated at 37 °C for 60 min. Subsequently, 75 μL of DNS reagent was added for the chromogenic reaction, and the mixture was boiled at 98 °C for 5 min to terminate the reaction. OD_530_ was detected in a 96-well plate using a Biotek EON microplate reader (Biotek, Santa Clara, CA, USA). To obtain the optimal reaction conditions for the TwCDA, we measured the enzyme activity at various temperatures (20, 25, 30, 35, 40, and 45 °C). Each experiment was performed in triplicate, and a statistical analysis was conducted with SPSS 26.0 (SPSS Inc., Chicago, IL, USA). The chemical formula prediction of the reaction products was detected by liquid chromatography combined with time-of-flight mass spectrometry (LC-coTOF-MS), with the inactivated crude enzyme group and blank group (i.e., (GlcNAc)_5_ aqueous solution) as the control groups.

### 2.9. Statistical Analysis

The SPSS version 26 software was used to analyze the experimental data statistically, and one-way analysis of variance was used for the data. The results were expressed as mean ± standard deviation (SD). *p* < 0.05 was considered a significant difference; *p* < 0.01 was considered an extremely significant difference.

## 3. Results

### 3.1. Extraction and Analysis of Chitin from T. weissflogii

**Figure 1:**
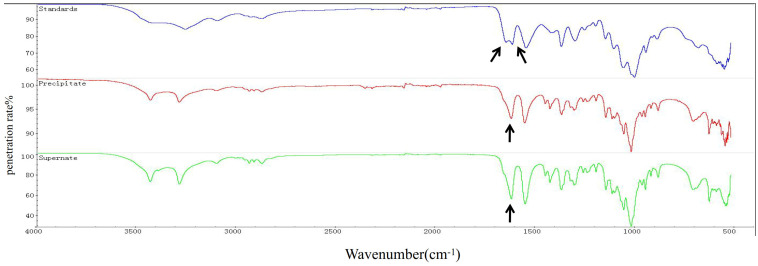
FTIR spectra of chitin (0–4000 cm^−1^). α-Chitin standard forms a double peak at 1620 cm^−1^ and 1652 cm^−1^ (the blue curve); the chitin extracted from the precipitation of *T. weissflogii* had a single peak at 1625 cm^−1^ (the red curve); the chitin extracted from the supernatant of *T. weissflogii* had a single peak at 1625 cm^−1^ (the green curve). The arrow indicates the location of the characteristic peaks.

An approximately 600 mL culture of *T. weissflogii* was used to extract chitin. The content of chitin obtained from the supernatant and cell pellets was 0.047 g and 0.0762 g, respectively. The total yield of chitin was calculated to be 20.53% of the total cell mass (fresh weight). Infrared spectrum detection showed that the chitin extracted from *T. weissflogii* had a single peak at 1625 cm^−1^, while α-chitin standard forms a double peak at 1620 cm^−1^ and 1652 cm^−1^ (Figure 1). The antiparallel sugar chains in α-chitin make the C=O bond stretch and vibrate, forming a hydrogen bond between C=O and the hydroxyl group and another hydrogen bond between C=O and N–H, whereas β-chitin can only form the former because of the parallel sugar segments [47]. Therefore, it was verified that the crystallographic structure of chitin produced by *T. weissflogii* is β-chitin.

### 3.2. Sequence Analysis of TwCDA

**Figure 2:**
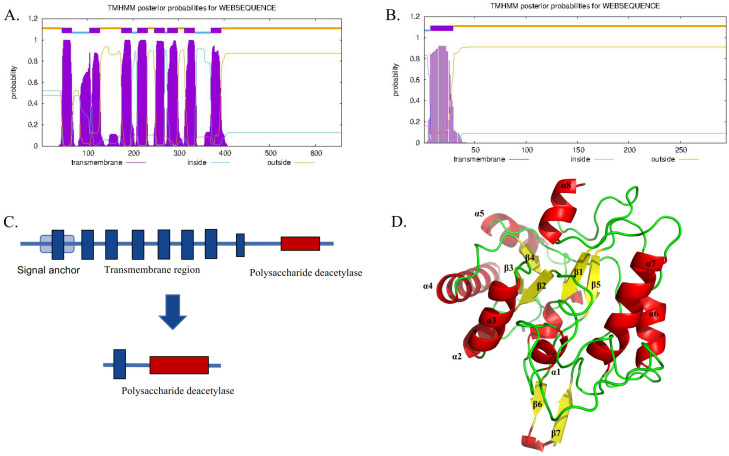
Sequence comparison between TwCDA and TwCDA-S. (**A**) TwCDA has 8 transmembrane helices at amino acids 44 to 393 and a signal anchor. (**B**) TwCDA-S has 1 transmembrane helix at amino acids 7 to 29. (**C**) Both TwCDA and TwCDA-S have a complete polysaccharide deacetylation motif at their *C*-terminal. (**D**) TwCDA’s tertiary structure. Stereo images of TwCDA and the canonical TIM barre (α/β)_8_ fold as observed in Tiny-TIM.

*TwCDA-S* is the *N*-terminal truncated sequence of *TwCDA* with only one transmembrane helical structure but retaining the complete polysaccharide deacetylation domain (Figure 2). The sequence features of TwCDA and TwCDA-S are summarized in Table 1. *TwCDA* (1974 bp) encoded 658 amino acids, which had a predicted molecular weight (MW) of 71.15 kDa and an isoelectric point (pI) of 6.21. *TwCDA-S* (885 bp) encoded 295 amino acids, with a predicted MW of 31.89 kDa and a pI of 5.11. TwCDA has a signal anchor, whereas TwCDA-S does not. Secondary structure prediction showed that both CDAs had a similar composition, with a predominance of the α-helix structure (~45%). The TwCDA tertiary structure reveals a single domain (residues 403–651) adopting a fold reminiscent of an (α/β)_8_ topology (Figure 2D), characteristic of the NodB homology domain present in all CE4 esterases [48].

**Figure 3:**
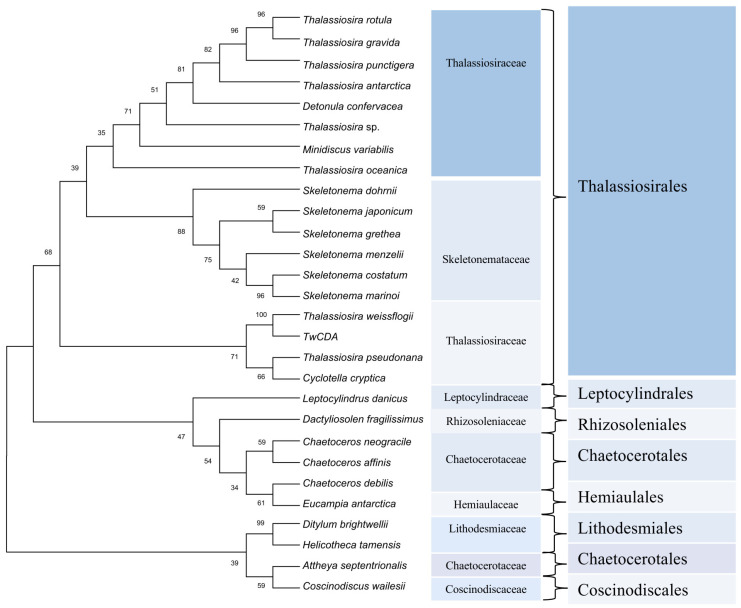
Phylogenetic tree constructed based on 28 putative chitin deacetylase amino acid sequences in diatoms. Proteins are from the MMETSP, PhycoCosm, and PLAZA databases. The tree was constructed using the neighbor-joining algorithm with 1000 bootstrap replicates. The number at each branch node represents a bootstrap value.

For the phylogenetic analysis, 93, 37, and 6 diatom sequences highly homologous to the full-length amino acid sequence of TwCDA were retrieved from the three available genome resources of the MMETSP [38], PhycoCosm [49], and PLAZA databases, respectively (Tables S2–S4). These sequences were highly enriched in Thalassiosirales (74.3%). In the 101 Thalassiosirales sequences, 81 (80.2%) were from Thalassiosiraceae and 17 (16.8%) from Skeletonemataceae. We arranged these 136 sequences according to sequence identity with TwCDA after BLASTP homologous alignment, and selected 28 CDA amino acid sequences with the highest similarity from each homologous species to illustrate the evolutionary history (Figure 3). The CDA sequences of these 28 CDAs are listed in Tables S5–S7. Among them, all the species of Thalassiosirales were clustered into one clade, and the CDAs from the genus *Skeletonema* were the most conserved, forming an independent group. Members of the genus *Thalassiosira* were distributed across different lineages within the Thalassiosirales. The CDA of genus *Chaetoceros* is more closely related to *Eucampia*, and *Leptocylindrus* and *Dactyliosolen* form individual branches. Figure 3 also shows that TwCDA was in the same node as the CDA from diatom *T. pseudonana* (NCBI Reference Sequence: XP_002293781.1) and *C. cryptica* (PLAZA Reference Sequence: CC00G25130). Multiple-sequence alignments indicated that TwCDA has 95% and 96% query coverage and 38.61% and 39.31% identity with *T. pseudonana* CDA and *C. cryptica* CDA, respectively (Figure S1). We also retrieved two CDA genes from a pennate diatom *Synedra acus*, with the amino acid sequences listed in Table S7.

### 3.3. Heterologous Expression and Deacetyl Activity of TwCDAs

**Figure 4:**
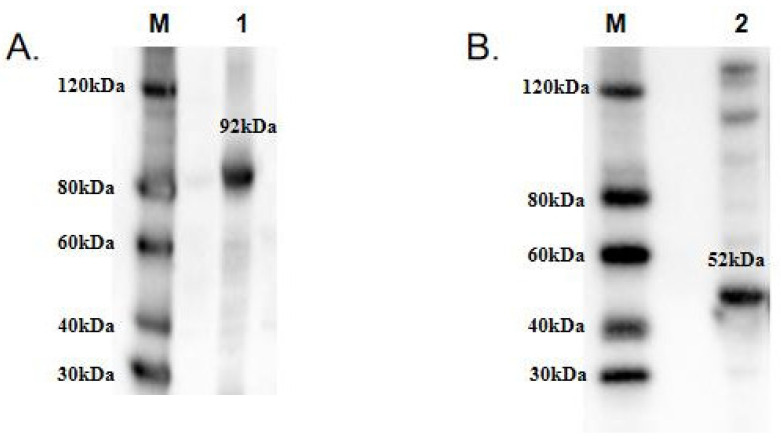
Western blot analysis of recombinant (**A**) TwCDA; (**B**) TwCDA-S. M: protein ladder; 1–2: TwCDA and TwCDA-S fusion proteins. To improve the clarity and conciseness of the presentation, these two gels have been cropped. Original Western blots are shown in Figure S3 (Supplementary Materials).

To functionally characterize TwCDAs and compare their enzyme characteristics, we induced large amounts of CDA expression. The protein concentrations of TwCDA and TwCDA-S were 3.1171 mg/mL and 3.374 mg/mL, respectively. The MWs of TwCDA and TwCDA-S fusion proteins were approximately 92 kDa and 52 kDa, respectively. The Western blot analysis confirmed the presence of proteins with the expected sizes (Figure 4 and Figure S2).

**Figure 5:**
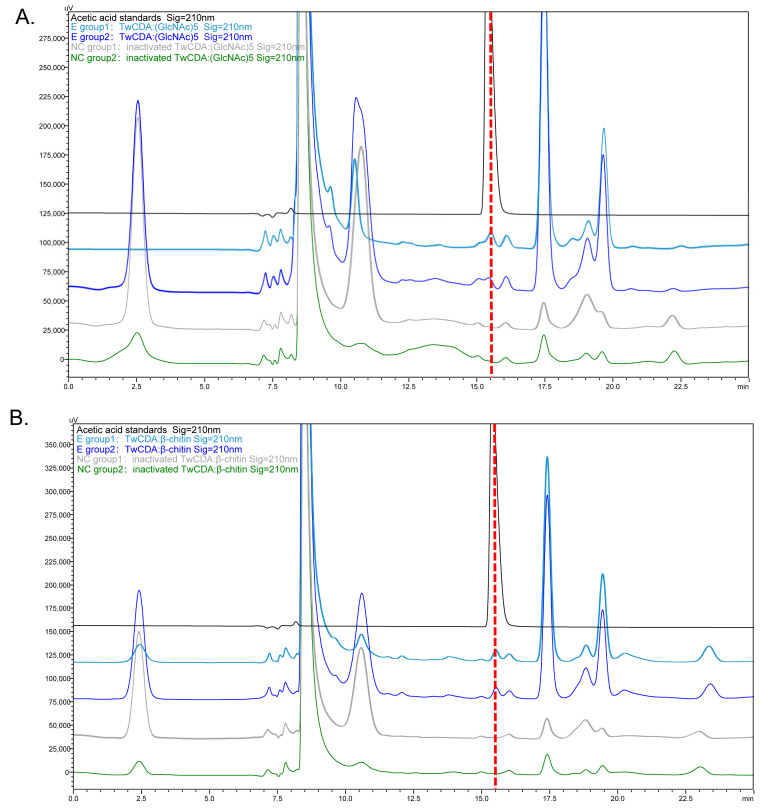
HPLC chromatograms presenting the deacetylation activity of TwCDA. (**A**) Chromatogram of TwCDA with (GlcNAc)_5_ as substrate. (**B**) Chromatogram of TwCDA with β-chitin as substrate. The retention time of acetic acid standard was 15.389 min (black curves). Acetic acid was produced in both experimental groups (dark blue and light blue curves) but not in the control groups (gray and green curves).

The deacetylation activities of TwCDA and TwCDA-S on (GlcNAc)_5_ and β-chitin were detected by HPLC (Figure 5 and Figure S3). The concentration of acetic acid produced in the reaction mixtures was calculated (Figure S3C). The HPLC analysis showed that TwCDA could catalyze the deacetylation of both (GlcNAc)_5_ (Figure 5A) and β-chitin (Figure 5B), and the catalyzation efficiency on the pentamer was 1.20 times higher than that on the polymer (Figure S3C). In contrast, TwCDA-S could not catalyze β-chitin deacetylation (Figure S3A,B), and the deacetylase activity against the oligosaccharide of (GlcNAc)_5_ was only 62.89% of that of TwCDA (Figure S3C). To summarize, TwCDA had higher deacetylase activity than TwCDA-S, and the activity was stronger when (GlcNAc)_5_ was used as a substrate. To further confirm whether acetic acid was produced in the reaction mixture, the products of TwCDA against the (GlcNAc)_5_ group were detected by GC-MS, and it was verified from the molecular weight of the characteristic peak that acetic acid (C_2_H_4_O_2_, MW = 60.02) was indeed produced in the mixture (Figure S4).

### 3.4. Chitinolytic Activity of TwCDA

**Figure 6:**
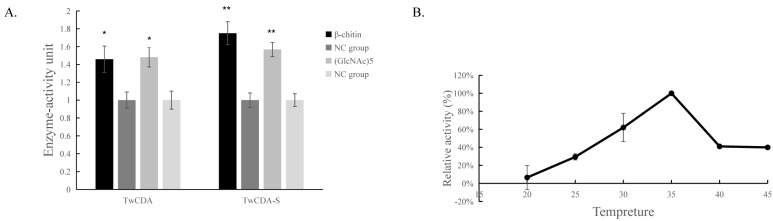
Chitinolytic activity of TwCDAs. (**A**) Enzymatic activities of TwCDA and TwCDA-S with β-chitin as substrate (black bar); enzymatic activities of TwCDA and TwCDA-S with (GlcNAc)_5_ as substrate (gray bar); control group (dark graybar and light gray bar). *, *p* < 0.05; **, *p* < 0.01. (**B**) Influence of temperature (20–45 °C) on the activities of TwCDA-S with (GlcNAc)_5_ as substrate. The error bars are the mean ± SD of three technical replicates for each sample.

**Figure 7:**
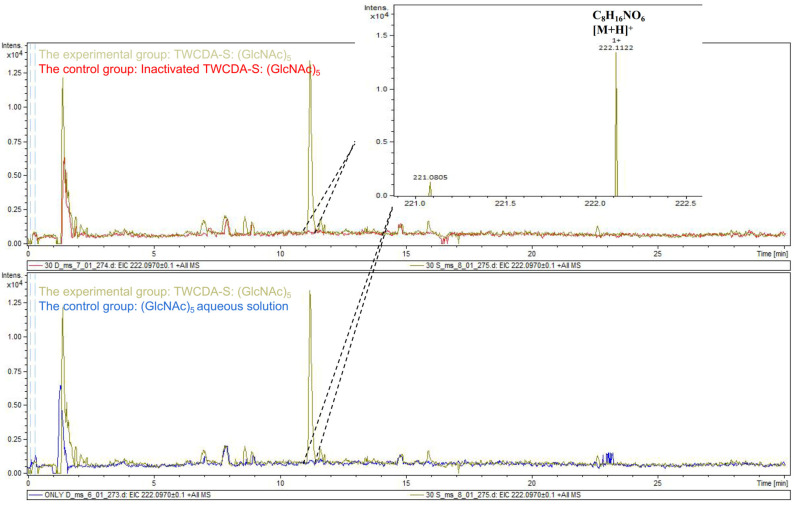
LC-coTOF-MS chromatograms. TwCDA-S and (GlcNAc)_5_ were taken as the experimental group (green curves), and inactivated TwCDA-S (above panel) and (GlcNAc)_5_ aqueous solution (below panel) were taken as the control group, respectively. The characteristic peak appeared at around 11 min in the experimental group, with the predicted molecular weight of 222.1122, which is in line with the MW of chitin monomer *N*-acetylglucosamine. Note the molecular ion species formed: [M + H]^+^.

The DNS assay showed that reducing sugars were produced in the products of the four experimental groups (TwCDA and TwCDA-S against pentamer and β-chitin). Taking the amount of enzyme required to produce 0.1 μmol NAG in 1 h as the unit of enzyme activity, we drew the specific activity bar chart with the number of units of enzyme activity per milligram of enzyme (Figure S5). We compared the difference in OD_530_ between the experimental group and the control group. The results indicated that regardless of whether β-chitin or (GlcNAc)_5_ was used as the substrate, TwCDA showed significantly higher chitinolytic activity than the control groups, with activity 1.48-fold (against pentamer) and 1.46-fold (against β-chitin) higher than the control groups (*p* < 0.05), respectively (Figure 6A). Compared with TwCDA, TwCDA-S presented higher chitinolytic activity on both (GlcNAc)_5_ and β-chitin, showing 1.57-fold and 1.75-fold higher activity than the control groups (*p* < 0.01), respectively (Figure 6A). This indicates that, without the transmembrane helix structure, TwCDA-S had higher deglycosylation activity. We then detected its chitinolytic activity at different temperatures with (GlcNAc)_5_ as the substrate, and found that the optimal reaction temperature was 35 °C (Figure 6B). It was found that TwCDA-S had the highest chitinolytic activity when (GlcNAc)_5_ was used as the substrate. All of the source data of the enzyme activity under different substrates and different temperatures are shown in Tables S8–S11. The molecular weight of the reaction product was analyzed by LC-coTOF-MS, with the inactivated TwCDA-S and (GlcNAc)_5_ solution as a control. A clear characteristic peak appeared at approximately 11 min in the experimental group, with a predicted molecular weight of 222.1122, which is in line with the MW of chitin monomer *N*-acetylglucosamine (C_8_H_15_NO_6_) (Figure 7).

## 4. Discussion

Diatoms represent an important source of β-chitin, which has been reported to be a better material for application compared with α-chitin [50]. β-Chitin displays higher reactivity for deacetylation and chemical modification due to its weak intermolecular interactions [7]. Although the biosynthesis and degradation pathway of chitin has been extensively investigated, especially in fungi and insects, there are few reports on chitin-relevant enzymes from β-chitin producers [36]. Chitosan and chitooligosaccharide are two of the most important derivatives and have great potential in the biomedical field. In their in vitro synthesis, chitin deacetylase (CDA) plays a crucial role in the enzymatic deacetylation of chitin polymers. At present, the research on the CDA mainly focuses on enzyme function analysis and crystal structure establishment [33,51]. In fungi and insects, CDAs may play a role in morphogenesis, whereas bacterial CDAs may contribute to chitin degradation for nutritional purposes [28,32]. In this study, we isolated a *CDA* gene from *T. weissflogii*, a high producer of β-chitin, and explored its structural and enzymatic properties.

Most CDAs have a conserved (α/β)_8_ barrel fold with six or seven strands and encompass a mononuclear metalloenzyme that employs a conserved His–His–Asp zinc-binding triad to carry out acid/base catalysis [33,51,52]. In this study, TwCDA had a characteristic of the NodB homology domain, adopting a fold reminiscent of an (α/β)_8_ topology, which is consistent with CDA structure studies. The full-length sequence contains 658 amino acids, but there are 403 amino acid residues in its *N*-terminal region, whose tertiary structure is not predicted, denoting the unstructured nature of this region [51]. The active site of the His–His–Asp zinc-binding triad was not found in TwCDA. Although Strunk et al. [53] reported that the conserved Asp–His–His triad was indispensable for a functional polysaccharide deacetylase, CDAs from diatoms seem to be an exception. This triad also disappeared in TpCDA (JGI Reference Sequence: Thaps3_J24880), which has been verified to catalyze the deacetylation of (GlcNAc)_5_ and the partially acetylated chitosan polymer [36]. Together with the detection of the deacetylase activity of the TwCDA in this study, we presumed that acid/base catalytic sites might be diversified and HHD is not conservative and indispensable in diatoms.

The putative CDA sequences from the MMETSP, PhycoSM, and PLAZA databases show that the CDAs are dominated by sequences from marine centric diatoms and the Thalassiosirales contain the most abundant CDA homologues. Sequence homology alignment found that CDAs were retrieved from two independent divisions: centric diatoms and pennate diatoms. In pennate diatoms, we first reported the presence of CDA homologous sequences in *Synedra acus*, which expanded the taxonomic distribution of CDA in diatoms and provided new sources of novel CDA enzymes. A phylogenetic tree found that TwCDA was closely related to *T. pseudonana* CDA and *C. cryptica* CDA. The evolutionary position of *C. cryptica* is adjacent to *T. pseudonana*. This was consistent with the finding that *T. pseudonana* likely descended from a freshwater ancestor in *Cyclotella* [54].

TwCDA was predicted to have a structure of eight transmembrane helices. The prediction of subcellular localization showed that TwCDA was potentially located in the endoplasmic reticulum. This was in accordance with the results of Shao et al. [36], who reported that the subcellular localization of *T. pseudonana* CDA–GFP fusion proteins is in the ER. These transmembrane helix structures may cause difficulties for our protein expression in vitro. However, producing a prokaryotic membrane protein in the simple, flexible, and inexpensive *E. coli* system has already proven successful in many cases [55,56,57]. Unfortunately, the yield and quality of membrane proteins, especially eukaryotic ones, are often insufficient for structural and functional studies in *E. coli* [58]. To address this issue, Wagner et al. [59] found that membrane protein production can be increased in *E. coli* through the rational design engineering of strains or by modifying target proteins. In addition, Brosson et al. [60] studied a CDA with transmembrane helices in the *Encephalitozoon cuniculi*, produced a recombinant form of the EcCDA protein that was slightly truncated to exclude transmembrane helices, and obtained a high yield of purified protein. Therefore, we removed as many transmembrane helices as possible on the basis of preserving the complete functional domain and expressed it in *E. coli*. We simultaneously expressed TwCDA with the full length of ORF and TwCDA-S with the transmembrane structure removed, and carried out enzyme activity detection. It was found that TwCDA-S had a higher expression level and higher chitinolytic activity. Although TwCDA retains its transmembrane helical structure, its expression level is acceptable and it has higher deacetylase activity.

We found that TwCDA-S had optimum chitinolytic enzyme activity at 35 °C. At present, the optimal conditions for the enzyme activity of chitinase have been studied in animals, plants, bacteria, and fungi. According to the previous results, the optimal temperature is 25–65 °C [61,62,63]. The optimum temperature of 35 °C measured in our study is within this range. This is consistent with the optimum temperature of CDAs from *Francisella tularensis* (35 °C) and *Coprinellus congregatus* (35 °C) [64,65].

Previous results have shown that most CDAs had high activity on chitooligosaccharides, water-soluble chitosan, and colloidal chitin, but low or no activity toward chitin polymer [30,66]. Research on chitin deacetylase has mostly used chitin oligosaccharide or chitosan as a substrate, but rarely chitin. Even in functional CDA studies in diatoms, diatom CDAs only had enzyme activities on partially deacetylated chitosan of DA20 and DA60, but there is no report of chitin polymers [36]. Chitin polymers are difficult to catalyze by CDA, due to the stable structure and insolubility. For example, Cord-Landwehr et al. [67] explored the activity of PesCDA from *Pestalotiopsis* sp., and revealed that this enzyme is inactive against insoluble α-chitin and β-chitin, slightly active against colloidal chitin, and active against soluble chitosan polymers. In our study, HPLC results revealed that TwCDA could catalyze both (GlcNAc)_5_ and β-chitin, but TwCDA-S can only catalyze (GlcNAc)_5_. We speculate that the *N*-terminal TMH or signal-anchoring sequence is essential for the catalysis of the polysaccharide. This speculation was supported by the previous report that a signal anchor is important for fungal CDAs to function efficiently at the cell surface [68]. The effective deacetylation of TwCDA on crystalline chitin substrates provides a new enzyme resource for producing chitin derivatives in vitro.

Most of the reports on CDAs are about its deacetylation activity; there have been few reports on its chitinolytic activity [46]. Chitinase activity can be divided into two types: exochitinase activity and endochitinase activity. Endochitinase activity randomly cleaves chitin at internal sites, whereas exochitinase acts at the end point of chitin oligosaccharides to liberate (GlcNAc)_2_ or GlcNAc [69]. Thus, the finding of a chitinolytic enzyme with exochitinase activity could be promising in regard to its potential for the production of GlcNAc by the enzymatic method [70]. Since we detected the presence of GlcNAc monosaccharide in the reaction mixture using LC-coTOF-MS, we speculated that TwCDA might display exochitinase activity. This makes TwCDA a more important candidate enzyme to be co-utilized to produce chitooligosaccharide, the most promising chitin derivative. However, glucosamine was not detected, presumably because TwCDA has no deacetylation activity on monosaccharide, which is consistent with previous studies showing that most CDAs have no deacetylation activity against GlcNAc monosaccharide [33,67,71]. However, our enzyme activity reaction was carried out for the crude enzyme solution, not the purified proteins. We need to further analyze the enzyme kinetic parameters on the basis of pure TwCDA for a deeper understanding of TwCDA.

In summary, a putative TwCDA gene was identified and characterized, and the catalytic products of TwCDA recombinant protein were explored. To our knowledge, this is the first analysis of chitin deacetylase with both chitin deacetylase and chitinase activities from diatoms, and the first report of a CDA that could catalyze β-chitin polymer. Although we used a crude TwCDA preparation instead of a pure enzyme in this study, it is worth mentioning that crude enzymes are often used in industry [72]. Therefore, TwCDA could be used to prepare chitosan in combination with partial chemical treatment or pretreated chitin, which needs to be carried out industrially in the near future.

## 5. Conclusions

In this study, a chitin deacetylase (TwCDA) from *Thalassiosira weissflogii* was heterologously expressed in vitro for functional analysis. The results showed that both the full-length sequence (TwCDA) and the *N*-terminal truncated sequence (TwCDA-S) had chitin deacetylase and chitinolytic activities after expression in *Escherichia coli*. TwCDA and TwCDA-S could catalyze the deacetylation of oligosaccharide (GlcNAc)_5_. TwCDA had higher deacetylase activity, and also catalyzed the deacetylation of the β-chitin polymer. TwCDA-S had high chitinolytic activity for (GlcNAc)_5_, and the optimal reaction temperature was 35 °C. Altogether, we isolated a chitin deacetylase from a marine diatom, which can catalyze the deacetylation and degradation of chitin and chitin oligosaccharides. The relevant results lay a foundation for the internal regulation mechanism of chitin metabolism in diatoms, and provide a candidate enzyme for the green industrial preparation of chitosan and chitin oligosaccharides. However, our enzyme activity reaction was carried out on the basis of a crude enzyme solution, and a sufficient quantity of active recombinant enzyme was not obtained after purification. We need to find solutions regarding how to retain the enzymatic activity during the purification process and further analyze the enzyme kinetic parameters for a deeper understanding of TwCDA.

## Figures and Tables

**Table 1:** Comparisons of TwCDA and TwCDA-S in terms of gene/protein structure, subcellular localization, and activities. CDS, coding sequences; ER, endoplasmic reticulum.

Name	TwCDA	TwCDA-S
Molecular weight (Kda)	71.15038	31.89025
Number of amino acids	658	295
Length of CDS (bp)	1974	885
Isoelectric point (pI)	6.21	5.11
Instability index	36.72	34.08
Signal peptide	0	0
Number of predicted TMHs	8	1
Conserved domain	Polysaccharide deacetylase	Polysaccharide deacetylase
Subcellular location	ER	-

## Data Availability

All data supporting the findings of this study are available in public databases, within the paper, and within its Supplementary Materials published online.

## Supplementary Materials

The following supporting information can be downloaded at: https://www.mdpi.com/article/10.3390/metabo13030429/s1, Table S1. Nutrient and metal concentration in optimized f/2 liquid medium. Table S2. Diatom sequences with high homology to the full-length amino acid sequences of TwCDA retrieved from PhycoCosm. Table S3. Diatom sequences with high homology to the full-length amino acid sequences of TwCDA retrieved from MMETSP. Table S4. Diatom sequences with high homology to the full-length amino acid sequences of TwCDA retrieved from PLAZA. Table S5. Diatom TwCDA homologous sequences retrieved in PhycoCosm Bacillariophyta database. Table S6. Diatom TwCDA homologous sequences retrieved in MMETSP database. Table S7. Diatom TwCDA homologous sequences retrieved in PLAZA diatoms 1.0 database. Table S8. Standard curves of different concentrations of *N*-acetylglucosamine and absorption value. Table S9. The source data of enzyme activity under different substrates. Table S10. The source data of enzyme activity under different temperatures. Table S11. The source data of SPSS of enzyme activity under different substrates. Figure S1. Multiple sequence alignment of TwCDA with *T. pseudonana* CDA (A) and *C. cryptica* CDA (B), respectively. Figure S2. Original Western blots of recombinant proteins. Figure S3. HPLC chromatograms and acetic acid standard curve. Figure S4. GC-MS of experimental group (above figure) and control group (below figure). Figure S5. Chitinolytic specific activity of TwCDAs. Enzymatic specific activities of TwCDA and TwCDA-S with β-chitin as substrate (black bar); enzymatic activities of TwCDA and TwCDA-S with (GlcNAc)_5_ as substrate (gray bar). The enzyme activities in the four experimental groups were significantly higher than those in the control group (dark gray bar and light gray bar). *, *p* < 0.05; **, *p* < 0.01.
