# Supplementary material for: Novel Chitin Deacetylase from Thalassiosira weissflogii Highlights the Potential for Chitin Derivative Production
Source: Metabolites. 2023 Mar 15;13(3):429. doi: 10.3390/metabo13030429 (PMC10057020; doi:10.3390/metabo13030429)
Supplement: Supplementary file 1 [file metabolites-13-00429-s001.zip › Supplementary Figures S1-S5.pdf]

A

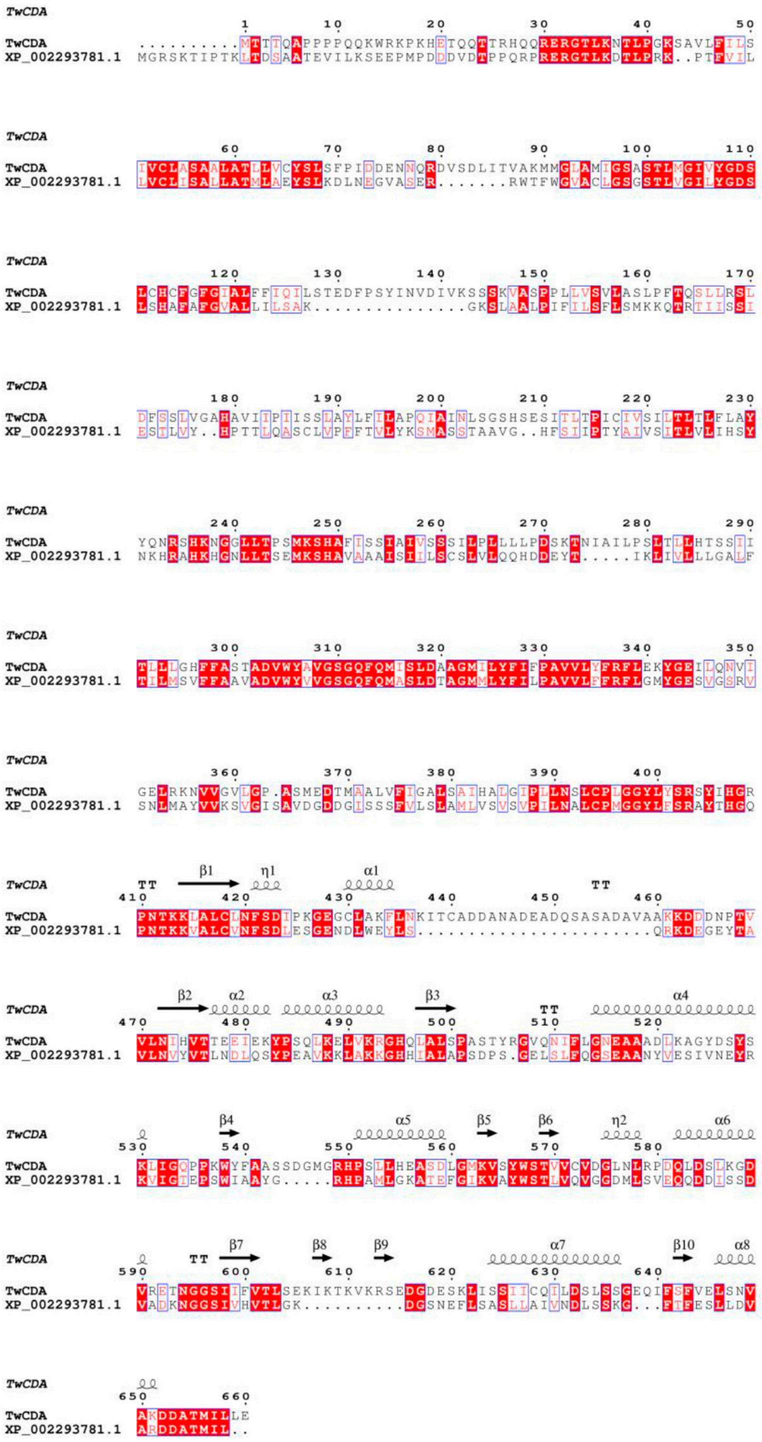

B

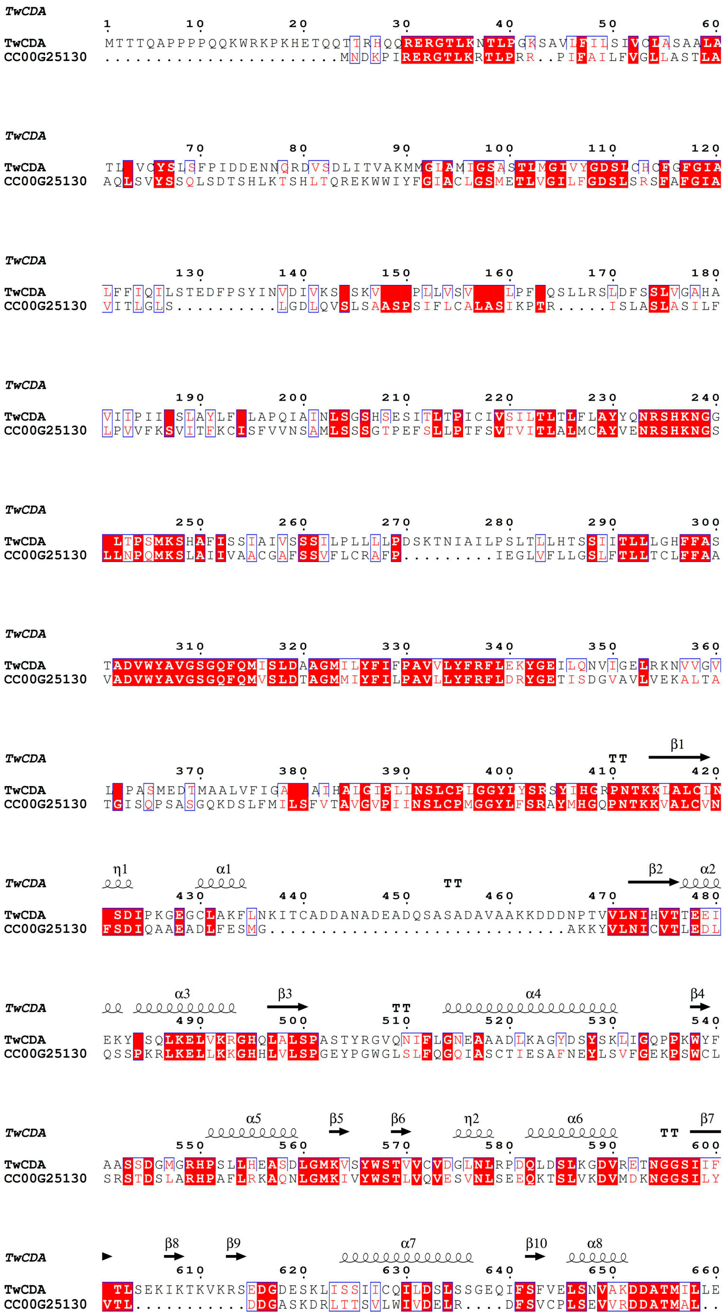

Figure S1. Multiple sequence alignment of TwCDA with T. pseudonana CDA (A) and C.cryptica CDA (B), respectively. Fully conserved residues are shaded red, TwCDA secondary structure elements are shown and labelled.

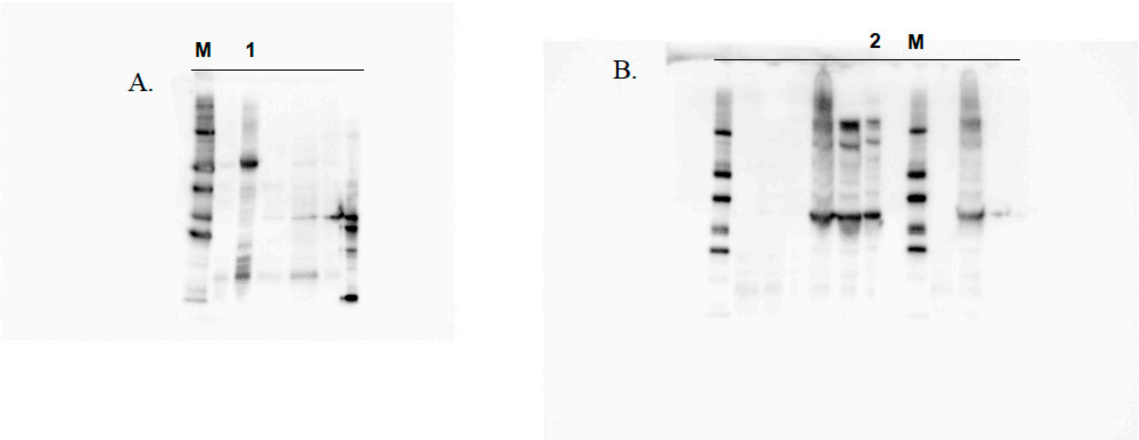

Figure S2. Original western blots of recombinant proteins. (A) TwCDA; (B) TwCDA-S. M: protein ladder; 1: TwCDA fusion protein; 2: TwCDA-S fusion proteins.

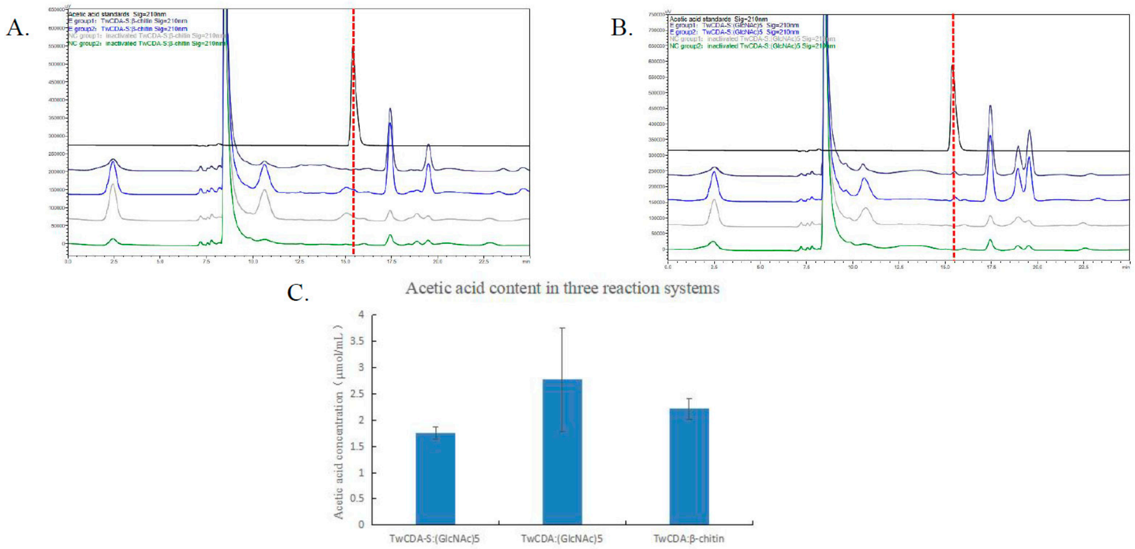

Figure. S3. HPLC chromatograms and acetic acid standard curve: (A) Chromatogram of TwCDA-S with $(GlcNAc)_5$ as substrate. Acetic acid was produced in both experimental groups (dark blue and light blue curves) but not in the control groups (gray and green curves). (B) Chromatogram of TwCDA-S with β-chitin as substrate. Acetic acid was not produced in the experimental groups (dark blue and light blue curves) and the control groups (gray and green curves). (C)The acetic acid concentration in the products of three reaction systems was calculated from the acetic acid standard curve equation. The error bars are the mean±SD of two technical replicates for each sample.

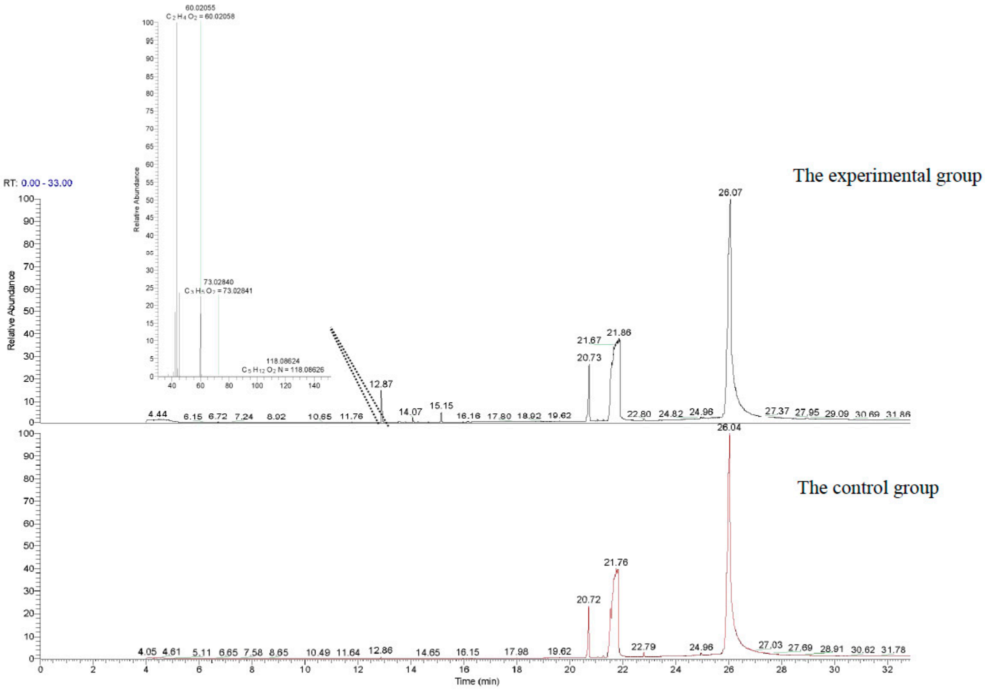

Figure S4. GC-MS of experimental group (above panel) and control group (below panel). Gas chromatogram showed that there was a characteristic peak at 12.87 min in the experimental group, and mass spectrometry revealed that the characteristic peak was generated by acetic acid ($C_2H_4O_2$).

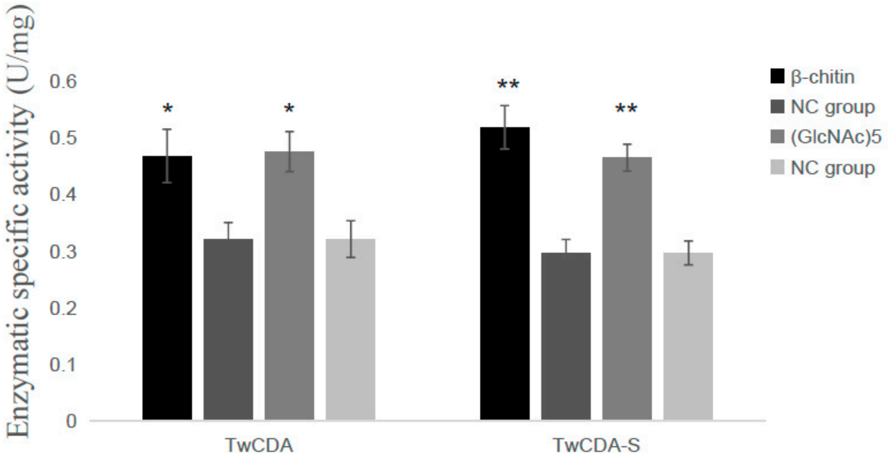

Figure S5. Chitinolytic specific activity of TwCDAs. (a) Enzymatic specific activities of TwCDA and TwCDA-S with β-chitin as substrate (black bar); Enzymatic activities of TwCDA and TwCDA-S with $(GlcNAc)_5$ as substrate (gray bar). The enzyme activities in the four experimental groups were significantly higher than those in the control group (dark gray bar and light gray bar). *, $P < 0.05$; **, $P < 0.01$.
